# Diagnostic reliability of clinical signs in cows with traumatic reticuloperitonitis and abomasal ulcers

**DOI:** 10.1186/s12917-020-02515-z

**Published:** 2020-09-29

**Authors:** Ueli Braun, Karl Nuss, Sonja Warislohner, Christina Reif, Carina Oschlies, Christian Gerspach

**Affiliations:** Department of Farm Animals, Vetsuisse-Faculty, University of Zurich, Winterthurerstrasse 260, CH-8057 Zurich, Switzerland

**Keywords:** Cattle, Traumatic reticuloperitonitis, Abomasal ulcus, Sensitivity, Specificity, Predictive values, Likelihood ratio_+_

## Abstract

**Background:**

Clinical signs of traumatic reticuloperitonitis and abomasal ulcer are often similar making the disorders difficult to differentiate. The goal of our study was to compare the frequency of individual clinical signs of cows with traumatic reticuloperitonitis and cows with abomasal ulcers and determine their diagnostic significance. The frequency of the findings “rectal temperature, heart rate, respiratory rate, demeanour, signs of colic, arched back, abdominal guarding, bruxism, scleral vessels, rumen motility, foreign body tests, percussion auscultation, swinging auscultation and faecal colour” of cows with traumatic reticuloperitonitis (TRP, *n* = 503) and cows with type 1 (U1, *n* = 94), type 2 (U2, *n* = 145), type 3 (U3, *n* = 60), type 4 (U4, *n* = 87) and type 5 (U5, *n* = 14) abomasal ulcer were compared, and the reliability indices “diagnostic sensitivity and specificity, positive and negative predictive values and positive likelihood ratio” were calculated. A total of 182 healthy cows served as controls (control group).

**Results:**

None of the cows in the control group had colic, rumen atony or melena, 99% had no abnormalities in demeanor and appetite and did not have a rectal temperature of ≤38.6 or >  40.0 °C, a heart rate >  100 bpm or a respiratory rate >  55 breaths per min, and 95% did not have an arched back or bruxism. The control group was therefore ideal for comparative purposes. Many signs such as mild increase in rectal temperature, scleral congestion and positive foreign body test were non-diagnostic because they occurred in healthy as well as in ill cows. Likewise, differentiation of cows with TRP and abomasal ulcer was not possible based on single clinical variables; a detailed history and a comprehensive assessment of all clinical findings were required for this.

**Conclusions:**

The findings of the present study serve as a guide for the veterinarian in the differentiation of cows with traumatic reticuloperitonitis and abomasal ulcer.

## Background

Traumatic reticuloperitonitis (TRP) and abomasal ulcer are important diseases, and together with displaced abomasum, belong to the most common gastrointestinal disorders of dairy cows. Traumatic reticuloperitonitis may be acute or chronic [1, 2] and abomasal ulcers are classified as types 1 (U1), 2 (U2), 3 (U3), 4 (U4) or 5 (U5) [3–8]. U1 is a non-perforated ulcer of the abomasal mucosa associated with minimal haemorrhage, whereas U2 involves the erosion of a large blood vessel and is therefore associated with massive intraluminal haemorrhage. U3 is a perforated ulcer accompanied by localised peritonitis, U4 is a perforated ulcer characterised by generalised peritonitis and U5 is an ulcer that has perforated into the omental bursa causing omental bursitis. Sometimes more than one type of abomasal ulcer occurs in the same cow [9]. The clinical findings of cows with TRP [10], U1 [11], U2 [12], U3 [13], U4 [14] and U5 [8] were recently described in detail, and overviews of older studies of cows with TRP [10] and of cows with abomasal ulcer [11] were also reported. A common thread running through all those studies was that the different disorders were often characterised by similar clinical manifestations [8, 10–14]. However, the frequency with which the clinical signs occured varied widely among the different disorders. The prognosis also varied and was usually favourable in cows with TRP [10] or U2 [12] but was hopeless in cows with U4 [14] or U5 [8]. Treatment of these diseases also differed, for example cows with TRP received antibiotics and a magnet [15], whereas recovery of cows with U2 usually required a blood transfusion [12]. If a correct diagnosis was possible based on the clinical examination, the reliability of the prognosis would increase allowing better decision-making with regard to treatment or euthanasia. To aid in the decision-making process, the frequencies of specific clinical findings were compared among cows with TRP, U1, U2, U3, U4 and U5, and the diagnostic sensitivity and specificity, the positive and negative predictive values and the likelihood ratio_+_ were calculated. These indices aid the clinician in differentiating the above-mentioned diseases and facilitate a diagnosis and the decision to treat or euthanase.

## Methods

### Animals

A total of 1085 cows including 182 healthy controls, 503 cows with TRP, 94 cows with U1, 145 cows with U2, 60 cows with U3, 87 cows with U4 and 14 cows with U5 were used. All animals were privately owned and transported to the Veterinary Teaching Hospital of the University of Zurich for clinical examination. The sample size differed between groups and was dictated by the case load, which varied depending of the incidence of the disease in the population.

### Tentative and definitive diagnosis of TRP and type 1, 2, 3, 4 or 5 abomasal ulcer

The tentative diagnosis of TRP was based on clinical findings that included abnormal general demeanour, fever, reduced rumen motility, positive foreign body test, poorly digested faeces and spontaneous signs of pain such as arching of the back, bruxism and grunting. The final diagnosis of TRP was based on radiographic evidence of a foreign body that penetrated or perforated the reticular wall or was seen outside of the reticulum (225 cattle), ultrasonographic changes of the reticular wall (403 cattle), laparoruminotomy and/or postmortem examination. In all cattle, the definitive diagnosis of TRP was based on more than one criterion. Cattle with TRP that had concomitant diseases causing anterior abdominal or caudal thoracic pain were excluded; this included 27 cows with bronchopneumonia and 24 cows with abomasal ulcers. In 449 of 470 cows (95.5%) with a complete history, the illness was acute with a maximum duration of 4 weeks and in 21 cows (4.5%) it was chronic with a duration of more than 4 weeks.

Type-1 ulcers were suspected in cows in the first weeks after calving with a poor appetite (non-specific indigestion characterised by fluctuations in appetite, moderate tympany, colic and dark, often soft to liquid faeces) when correction of possible underlying disorder such as displaced abomasum did not have the expected result. The final diagnosis of U1 was based on the results of laparotomy, when focal thinning of the abomasal wall was seen or an ulcer could be palpated, and/or finding an ulcer at postmortem examination. Cows in which U1 was accompanied by U2, U3, U4 or U5 were excluded from the study.

The diagnosis of U2 was based on typical manifestations of intraluminal haemorrhage in the anterior gastrointestinal tract including tachycardia, pale mucous membranes, dark or black manure, low haematocrit and the presence of blood in the faeces when abomasal volvulus, intussusception, haemorrhagic bowel syndrome, haemorrhage in the pharyngeal region and pulmonary haemorrhage could be ruled out as the cause of blood loss. In 100 of 103 (97%) cows, a test to detect occult blood in the faeces was positive. In seven cows, exploratory right-flank laparotomy was necessary to make a diagnosis. The diagnosis was confirmed at postmortem examination in cows that died or were euthanased.

Type-3 abomasal ulcer was suspected in cows with nonspecific clinical signs resembling TRP when other diseases associated with localised peritonitis such as TRP could be ruled out by radiography and ultrasonography. A final diagnosis of U3 was made when exploratory laparotomy showed fibrinous adhesions between the abomasum and the peritoneum and a reticular foreign body could be ruled out, or when the ulcer was detected at postmortem examination.

A tentative diagnosis of U4 was made in cows with clinical signs associated with generalised peritonitis such as tachycardia, tachypnoea, fever, congested scleral vessels, pale and muddy mucous membranes, decreased skin surface temperature, spontaneous grunting, abdominal guarding, diarrhea and reduction in the normally negative intraabdominal pressure on rectal examination. The final diagnosis of U4 was based in all cows on the results of postmortem examination.

A tentative diagnosis of U5 was made in cows with clinical signs of subacute to chronic peritonitis such as obtunded demeanour, indigestion, abdominal guarding, rumen atony and fever and ultrasonographic evidence of localised or generalised peritonitis. The final diagnosis of U5 was based on the results of postmortem examination when a perforated abomasal ulcer accompanied by omental bursitis was seen.

For U2, U3, U4, U5: No cases were excluded.

The control cows consisted of 182 healthy cows that were the offspring of cows with BSE and therefore referred by the Federal Veterinary Office BVET Switzerland to our clinic for examination. All the cows had a history of being healthy and were examined and monitored for demeanour, appetite, urination and defecation for several days. Although several cows had one abnormality, such as increased rectal temperature, increased heart rate, scleral congestion or reduced rumen motility, they were considered healthy based on all the variables monitored daily.

There were no other inclusion or exclusion criteria for including animals in the study.

### Treatment and response to treatment

Initial treatment of 503 cows with TRP was selected on the basis of radiographic findings at the time of admission [15]. Cattle with a foreign body attached to a magnet were treated conservatively (amoxicillin or penicilline G procaine, slow intravenous drip infusion of a solution containing sodium chloride and glucose), and cattle with radiographic evidence of a foreign body (non-penetrating, penetrating or perforating) were treated with antibiotics and a magnet. Radiographic examination was repeated on the following day and the results were used to guide subsequent treatment. Conservative treatment was continued when the foreign body was completely attached to the magnet and another radiograph was made. In cases where the foreign body was not in contact with the magnet or was still penetrating or perforating the reticulum, rumenotomy was carried out. Of 232 cattle, 191 (82%) were treated successfully and discharged and 41 did not respond to treatment and were euthanased. Surgical treatment was successful in 186 (90%) of 206 cases.

Seventy-eight (83%) cows with U1 were euthanased immediately after the initial examination, during laparotomy or after unsuccessful treatment (correcting of the main clinical problem, intravenous administration of a solution containing sodium chloride and glucose, flunixin meglumine or metamizole), and eight (8.5%) cows died, and all underwent a postmortem examination [11]. Eight (8.5%) cows were discharged and six of these made a complete recovery.

Ten of 145 cows with U2 were euthanased immediately after, or died during, the initial examination, and treatment was started in 135 cows [12]. Treatment included blood transfusion, sodium chloride and glucose solution, calcium borogluconate, vitamin C and metamizole. Ninety-one (67%) of the 135 treated cows recovered and were discharged after a mean hospitalisation period of 9 days, and 44 cows (33%) failed to respond to treatment and were euthanased or died.

Forty-eight of 60 (80%) cows with U3 were euthanased immediately after the initial examination, during laparotomy or after unsuccessful treatment (solution of sodium chloride and glucose administered via an indwelling jugular catheter, antibiotics, metamizole or flunixin) [13]. Twelve (20%) cows, that were treated, were discharged from the clinic.

Fifty-four of 87 (62%) cows in which U4 was diagnosed were euthanased immediately except for a few that died during the examination [14]. Twelve (14%) cows underwent right flank exploratory laparotomy to confirm the diagnosis and all were euthanased because of generalised peritonitis. The cows with an unclear diagnosis received a continuous intravenous infusion of a sodium chloride and glucose solution, antibiotics and an NSAID. Six cows died after the start of treatment and the remaining 15 were euthanased after 2 to 4 days because of deterioration in condition.

Eight of 14 (57%) cows with U5 were treated (see U4) unsuccessfully and euthanased thereafter [8]. Six cows were euthanased immediately after the initial examination.

### Euthanasia

Euthanasia was done with pentobarbital (Esconarkon, Streuli Pharma), 80 mg/kg body weight intravenously.

### Analysis of clinical variables

The clinical findings of the controls [16] and the cows with TRP [10], U1 [11], U2 [12], U3 [13], U4 [14]) and U5 [8] have been described in detail. The variables demeanour, rectal temperature, heart rate, respiratory rate, colic, arched back, abdominal guarding, mucous membrane colour, congestion of scleral vessels, bruxism, rumen motility, pain response to foreign body tests, positive percussion and simultaneous auscultation also called percussion auscultation (produces a tympanic sound or metallic ping) and ballottement and simultaneous auscultation also referred to as swinging auscultation (produces splashing sounds followed by a bell-like echo) on the right side and faecal colour were analysed. The following reference intervals, defined previously [17] and modified slightly, were used for the metric variables: rectal temperature, 38.6–39.0 °C; heart rate, 60–80 beats/min (bpm) and respiratory rate, 15–35 breaths/min.

### Statistical analysis

The program SPSS Version 25 was used. The Shapiro-Wilk test was used to test the metric variables rectal temperature and heart and respiratory rates for normality. Because the data were non-normal, the medians and 25th and 75th percentiles were calculated. The medians underwent one-factor analysis of variance and pair-wise comparison using the Kruskal-Wallis test. Frequency distributions of all variables were calculated for the controls and 6 disease groups. The values of all variables were divided into appropriate ranges (for instance the rectal temperature; < 38.5 °C, 38.6 to 39.0 and >  39.0 °C) and differences between ranges were analysed using the chi-square and the Bonferroni post-hoc tests. A *P*-value < 0.05 was considered significant. For each variable, the diagnostic sensitivity (a/[a + c]), the diagnostic specificity (d/[b + d]), the positive likelihood ratio (LR_+_, sensitivity/[1-specificity]), the positive predictive value (a/[a + b]) and the negative predictive value (d/[c + d]) were calculated (a, true positive; b, false positive; c, false negative; d, true negative) [18]. A false positive result was an abnormal finding in a control cow and a false negative result was a normal finding in a cow with TRP or abomasal ulcer.

## Results

### Rectal temperature

**Fig. 1:**
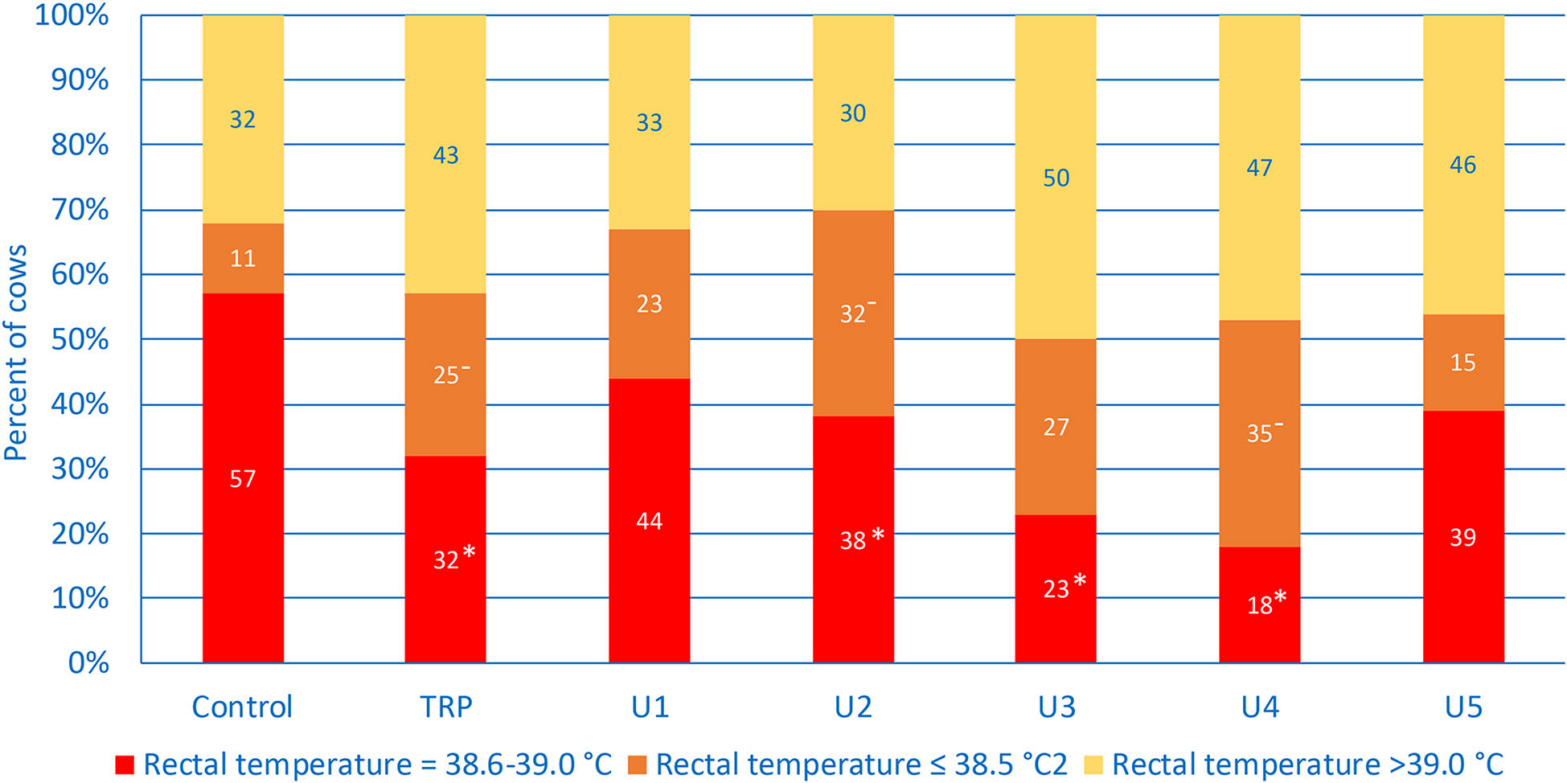
Rectal temperature in healthy control cows and cows with traumatic reticuloperitonitis (TRP), type 1 (U1), type 2 (U2), type 3 (U3), type 4 (U4) and type 5 (U5) abomasal ulcer. Frequency distribution of cows with a rectal temperature within, below or above the reference interval. * Different from percentage of controls in the reference interval, *P* < 0.05, ^−^ different from percentage of controls below the reference interval, *P* < 0.05

The median rectal temperatures ranged from 38.9 to 39.1 °C and did not differ among groups (Table 1). The rectal temperature was in the reference interval (38.6 to 39.0 °C, Table 2, Fig. 1) in 57% of the controls. With the exception of cows with U1 and U5, there were significantly fewer ill cows with a rectal temperature in the reference interval compared with controls. Eleven percent of controls had a temperature below the reference interval, and in 1% the temperature was ≤38.0 °C. Significantly more cows with TRP (25%), U2 (32%) and U4 (35%) had a rectal temperature ≤ 38.5 °C compared with controls. The LR_+_ for a rectal temperature ≤ 38.0 °C was 14 for cows with U2 and U5 and 17 for cows with U4 (Table 3). Rectal temperatures > 39.0, > 39.5 and >  40.0 °C occurred in 32, 6 and 1% of controls, and 23% of cows with U4 had a rectal temperature >  39.5 °C, which was significantly different from the controls.

**Table 1:** Rectal temperature and heart and respiratory rates in healthy control cows, cows with traumatic reticuloperitonitis (TRP) and cows with type 1 (U1), type 2 (U2), type 3 (U3), type 4 (U4) or type 5 (U5) abomasal ulcer (medians, 25 and 75% percentiles in brackets)

Variable	Controls (*n* = 182)	TRP (*n* = 500)	U1 (*n* = 93)	U2 (*n* = 144)	U3 (*n* = 60)	U4 (*n* = 86)	U5 (*n* = 13)
Rectal temperature (°C)	38.9 (38.7–39.2)	39.0 (38.5–39.3)	38.9 (38.6–39.2)	38.9 (38.3–39.1)	39.1 (38.5–39.4)	38.9 (38.3–39.5)	38.9 (38.6–39.2)
Heart rate (beats/min)	72 (64–80)	76^1^ (68–84)	80^1,2^ (68–101)	108^1,2,3,5^ (96–122)	84^1,2^ (76–99)	99^1,2^ (76–116)	76 (69–90)
Respiratory rate (breaths/min)	27 (24–32)	28 (24–32)	28^1^ (20–36)	29^1^ (24–36)	28 (24–40)	32^1^ (24–41)	29 (24–38)

^1–5^, *P* < 0.05, Kruskal Wallis test

^1^ Different from controls

^2^ Different from TRP

^3^ Different from U1

^4^ Different from U2

^5^ Different from U3

**Table 2:** Frequency distributions of the clinical findings in healthy control cows, cows with traumatic reticuloperitonitis (TRP) and cows with type 1 (U1), type 2 (U2), type 3 (U3), type 4 (U4) or type 5 (U5) abomasal ulcer

Variable	Classification	Controls	TRP	U1	U2	U3	U4	U5	Chi^2^
Rectal temperature	38.6–39.0 °C (normal range) ≤ 38.5 or > 39.0 °C	103 (57%)^2,4,5,6^ 79 (43%)	160 (32%)^1^ 343 (68%)	41 (44%)^6^ 52 (56%)	56 (38%)^1,6^ 89 (61%)	14 (23%)^1^ 46 (77%)	16 (18%)^1,3,4^ 71 (82%)	5 (39%) 8 (62%)	79
	≤ 38.5 °C (decreased)	21 (11%)^2,4,6^	126 (25%)^1^	21 (23%)	46 (32%)^1^	16 (27%)	30 (35%)^1^	2 (15%)	
	> 38.5 °C	161 (89%)	377 (75%)	72 (77%)	99 (68%)	44 (73%)	57 (66%)	11 (85%)	26
	≤ 38.0 °C (decreased)	2 (1%)^3,4,6,7^	34 (7%)^4,6^	8 (9%)^1^	22 (15%)^1, 2^	4 (7%)	16 (18%)^1, 2^	2 (15%)^1^	
	> 38.0 °C	180 (99%)	469 (93%)	85 (91%)	123 (85%)	56 (93%)	71 (82%)	11 (85%)	36
	> 39.0 °C (increased)	58 (32%)	217 (43%)	31 (33%)	43 (30%)	30 (50%)	41 (47%)	6 (46%)	
	≤ 39.0 °C	124 (68%)	286 (57%)	62 (67%)	102 (70%)	30 (50%)	46 (53%)	7 (54%)	20
	> 39.5 °C (increased)	11 (6%)^6^	71 (14%)^4^	9 (10%)	6 (4%)^2,6^	11 (18%)^4^	20 (23%)^1,4^	0 (0%)	
	≤ 39.5 °C	171 (94%)	432 (86%)	84 (90%)	139 (96%)	49 (82%)	67 (77%)	13 (100%)	30
	> 40.0 °C (increased)	1 (1%)	26 (5%)	3 (3%)	2 (1%)	6 (10%)	3 (3%)	0 (0%)	
	≤ 40.0 °C	181 (99%)	477 (95%)	90 (97%)	143 (99%)	54 (90%)	84 (97%)	13 (100%)	14
Heart rate	60–80 bmp (normal range) < 60 or > 80/min	139 (76%)^3,4,5,6^ 43 (24%)	349 (70%)^3,4,5,6^ 153 (30%)	45 (48%)^1,2,4^ 49 (52%)	15 (10%)^1,2,3,5,6,7^ 130 (90%)	27 (45%)^1,2,4^ 33 (55%)	26 (30%)^1,2,4^ 61 (70%)	8 (57%)^4^ 6 (43%)	220
	> 80/min (increased)	28 (15%)^3,4,5,6^	130 (26%)^3,4,5,6^	46 (49%)^1,2,4^	130 (90%)^1,2,3,5,6,7^	33 (55%)^1,2,4^	59 (68%)^1,2,4^	5 (36%)^4^	
	≤ 80/min	154 (85%)	372 (74%)	48 (51%)	15 (10%)	27 (45%)	28 (32%)	9 (64%)	274
	> 100/min (increased)	2 (1%)^3,4,5,6,7^	22 (4%)^3,4,5,6,7^	23 (24%)^1,2,4,7^	90 (62%)^1,2,3,5,6^	10 (17%)^1,2,4,6,7^	36 (41%)^1,2,4,5,7^	12 (86%)^1,2,3,5,6^	
	≤ 100/min	180 (99%)	480 (96%)	71 (75%)	55 (38%)	50 (83%)	51 (59%)	2 (14%)	368
	> 120/min (increased)	1 (1%)^3,4,6^	7 (1%)^3,4,6^	7 (7%)^1,2,4^	37 (26%)^1,2,3,5^	1 (2%)^4^	13 (15%)^1,2^	1 (7%)	
	≤ 120/min.	181 (99%)	495 (99%)	87 (93%)	108 (74%)	59 (98%)	74 (85%)	13 (93%)	137
Respiratory rate	15–35 breaths/min (normal range) < 15 or > 35/min	155 (85%)^4,5,6^ 27 (15%)	380 (76%) 121 (24%)	67 (71%) 27 (29%)	92 (64%)^1^ 52 (36%)	38 (63%)^1^ 22 (37%)	53 (62%)^1^ 33 (38%)	9 (64%) 5 (36%)	31
	> 35/min (increased)	27 (15%)^2,3,4,5,6,7^	117 (23%)^1,4,5,6^	27 (29%)^1^	52 (36%)^1,2^	22 (37%)^1,2^	33 (38%)^1,2^	5 (36%)^1^	
	≤ 35/min	155 (85%)	384 (77%)	67 (71%)	92 (64%)	38 (63%)	53 (62%)	9 (64%)	33
	> 45/min (increased)	4 (2%)^3,4,5,6,7^	21 (4%)^3,4,5,6,7^	13 (14%)^1,2,7^	20 (14%)^1,2,7^	9 (15%)^1,2,7^	17 (20%)^1,2,7^	14 (100%)^1,2,3,4,5,6^	
	≤ 45/min	178 (98%)	480 (96%)	81 (86%)	124 (86%)	51 (85%)	69 (80%)	0 (0%)	186
	> 55/min (increased)	1 (1%)^3,4,7^	12 (2%)^3,4,7^	9 (10%)^1,2^	12 (8%)^1,2^	4 (7%)	14 (16%)^1,2^	0	
	≤ 55/min	181 (99%)	489 (98%)	85 (90%)	132 (92%)	56 (93%)	72 (84%)	0	47
Demeanour	abnormal	2 (1%)^2,3,4,5,6,7^	438 (87%)^1,4,6^	88 (94%)^1,6^	141 (98%)^1,2^	57 (95%)^1,6^	87 (100%)^1,2,3,5,7^	13 (93%)^1,6^	
	normal	180 (99%)	65 (13%)	6 (6%)	4 (2%)	3 (5%)	0 (0%)	1 (7%)	690
Signs of colic	yes	0 (0%)^3,4,6,7^	16 (3%)^3,4,6^	20 (21%)^1,2,5^	15 (10%)^1.2^	1 (2%)^3^	9 (10%)^1,2^	2 (14%)^1^	
	no	182 (100%)	487 (97%)	74 (79%)	130 (90%)	59 (98%)	78 (90%)	12 (86%)	71
Arched back	yes	1 (0.5%)^2,3,5,6,7^	91 (18%)^1,4^	11 (12%)^1^	5 (3%)^2,6,7^	8 (13%)^1^	24 (28%)^1,4^	5 (36%)^1,4^	
	no	181 (99%)	412 (82%)	83 (88%)	140 (97%)	52 (87%)	63 (72%)	9 (64%)	69
Abdominal guarding	yes no	36 (20%)^2,3,4,5,6,7^ 146 (80%)	273 (54%)^1,6,7^ 228 (46%)	53 (59%)^1,6^ 37 (41%)	61 (43%)^1,6,7^ 81 (57%)	36 (61%)^1^ 23 (39%)	68 (81%)^1,2,3,4^ 16 (19%)	14 (100%)^1,2,4^ 0 (0%)	125
Bruxism	yes	1 (0.5%)^2,3,4,5,6,7^	103 (21%)^1^	14 (15%)^1^	18 (12%)^1^	11 (18%)^1^	22 (25%)^1^	3 (21%)^1^	
	no	181 (99.5%)	400 (79%)	80 (85%)	127 (88%)	49 (82%)	65 (75%)	11 (79%)	48
Scleral vessels	congested	42 (29%)^2,3,5,6,7^	393 (79%)^1,4^	83 (89%)^1,4^	51 (35%)^2,3,5,6,7^	43 (73%)^1,4^	66 (77%)^1,4^	11 (79%)^1,4^	
	not congested	104 (71%)	107 (21%)	10 (11%)	94 (65%)	16 (27%)	20 (23%)	3 (21%)	216
Mucous membranes	pale	5 (3%)^4,6^	37 (7%)^4,6^	9 (10%)^4^	100 (69%)^1,2,3,5,6,7^	2 (3%)^4,6^	23 (27%)^1,2,4,5^	2 (14%)^4^	
	not pale	177 (97%)	458 (93%)	83 (90%)	45 (31%)	58 (97%)	62 (73%)	12 (86%)	360
Rumen motility	reduced or absent	24 (13%)^2,3,4,5,6,7^	354 (73%)^1,4,5,6^	54 (90%)^1^	131 (92%)^1,2^	53 (93%)^1,2^	85 (93%)^1,2^	13 (93%)^1^	
	normal	158 (87%)	133 (27%)	6 (10%)	12 (8%)	4 (7%)	1 (7%)	1 (7%)	372
	absent (rumen atony)	0 (0%)^2,3,4,5,6,7^	29 (6%)^1,3,4,5,6,7^	50 (56%)^1,2,6^	64 (45%)^1,2^	28 (49%)^1,2^	23 (27%)^1,2,3^	5 (36%)^1,2^	
	reduced or normal	182 (100%)	458 (94%)	40 (44%)	79 (55%)	29 (51%)	63 (73%)	9 (64%)	278
Foreign body tests	at least one test positive	81 (44%)^2,3^	296 (60%)^1,3,4^	19 (24%)^1,2,6^	36 (28%)^2,6^	26 (45%)	44 (58%)^3,4^	5 (39%)	
	all foreign body tests negative	101 (56%)	199 (40%)	61 (76%)	91 (72%)	32 (55%)	32 (42%)	8 (61%)	70
PSA/BSA on the right side	PSA and/or BSA positive	19 (10%)^3,4,5,6,7^	87 (17%)^3,4,5,6,7^	50 (53%)^1,2,4^	47 (33%)^1,2,3^	28 (47%)^1,2^	44 (51%)^1,2^	9 (64%)^1,2^	
	PSA and BSA negative	163 (90%)	416 (83%)	44 (47%)	96 (67%)	32 (53%)	43 (49%)	5 (36%)	133
Faecal colour	dark or black	0 (0%)^3,4,5,6,7^	0 (0%)^3,4,5,6,7^	14 (18%)^1,2,4^	115 (80%)^1,2,3,5,6,7^	6 (11%)^1,2,4^	12 (16%)^1,2,4^	3 (21%)^1,2,4^	
	normal	182 (100%)	486 (100%)	64 (82%)	28 (20%)	47 (89%)	63 (84%)	11 (79%)	615

*PSA* Percussion and simultaneous auscultation, *BSA* Ballottement and simultaneous auscultation, *NR* Not recorded, *NA* Not applicable

^1^ Different from controls, *P* < 0.01

^2^ Different from TRP, *P* < 0.01

^3^ Different from U1, *P* < 0.01

^4^ Different from U2, *P* < 0.01

^5^ Different from U3, *P* < 0.01

^6^ Different from U4, *P* < 0.01

^7^ Different from U5 *P* < 0.01

**Table 3:** Diagnostic sensitivity and specificity, positive and negative predictive values and positive likelihood ratio of clinical findings in cows with traumatic reticuloperitonitis (TRP) and cows with type 1 (U1), type 2 (U2), type 3 (U3), type 4 (U4) or type 5 (U5) abomasal ulcer (in percent)

Variable	Reliability indices	TRP	U1	U2	U3	U4	U5
Rectal temperature ≤ 38.5 °C	Diagn. sensitivity	25	29	31	27	34	15
	Diagn. specificity	*88*	*88*	*88*	*88*	*88*	*88*
	Pos. pred. Value	*86*	50	68	43	59	9
	Neg. pred. Value	30	69	62	79	74	**94**
	LR_+_	2	2	3	2	3	1
Rectal temperature ≤ 38.0 °C	Diagn. sensitivity	7	9	15	7	18	15
	Diagn. specificity	**99**	**99**	**99**	**99**	**99**	**99**
	Pos. pred. Value	**94**	*80*	**92**	67	*89*	50
	Neg. pred. Value	28	68	59	76	72	**94**
	LR_+_	6	8	***14***	6	***17***	***14***
Rectal temperature > 39.0 °C	Diagn. sensitivity	43	33	30	50	47	50
	Diagn. specificity	68	68	68	68	68	68
	Pos. pred. Value	79	35	43	34	41	9
	Neg. pred. Value	30	67	55	*81*	73	**95**
	LR_+_	1	1	1	2	1	2
Rectal temperature > 39.5 °C	Diagn. sensitivity	14	10	4	18	23	NA
	Diagn. specificity	**94**	**94**	**94**	**94**	**94**	**94**
	Pos. pred. Value	*87*	45	35	50	65	NA
	Neg. pred. Value	28	67	55	78	72	**93**
	LR_+_	2	1	1	3	4	NA
Rectal temperature > 40.0 °C	Diagn. sensitivity	5	3	1	10	3	NA
	Diagn. specificity	**99**	**99**	**99**	**99**	**99**	**99**
	Pos. pred. Value	**96**	75	67	*86*	75	NA
	Neg. pred. Value	28	67	56	77	68	**93**
	LR_+_	9	6	3	***18***	6	NA
Heart rate > 80 bpm	Diagn. sensitivity	26	49	**90**	55	68	36
	Diagn. specificity	*85*	*85*	*85*	*85*	*85*	*85*
	Pos. pred. Value	*82*	62	*82*	54	68	15
	Neg. pred. Value	29	76	**91**	*85*	*85*	**94**
	LR_+_	2	3	6	4	4	2
Heart rate > 100 bpm	Diagn. sensitivity	4	24	62	17	41	*86*
	Diagn. specificity	**99**	**99**	**99**	**99**	**99**	**99**
	Pos. pred. Value	**92**	**92**	**98**	*83*	**95**	*86*
	Neg. pred. Value	27	72	77	78	78	**99**
	LR_+_	4	***22***	***56***	***15***	***38***	***78***
Heart rate > 120 bpm	Diagn. sensitivity	1	7	26	2	15	7
	Diagn. specificity	**99**	**99**	**99**	**99**	**99**	**99**
	Pos. pred. Value	*88*	*88*	**97**	50	**93**	50
	Neg. pred. Value	27	68	63	75	71	**93**
	LR_+_	3	***14***	***46***	3	***27***	***13***
Respiratory rate > 35 breaths/min	Diagn. sensitivity	23	29	36	37	38	36
	Diagn. specificity	*85*	*85*	*85*	*85*	*85*	*85*
	Pos. pred. Value	*81*	50	66	45	55	16
	Neg. pred. Value	29	70	63	*80*	75	**95**
	LR_+_	2	2	2	2	3	2
Respiratory rate > 45 breaths/min	Diagn. sensitivity	4	14	14	15	20	NA
	Diagn. specificity	**98**	**98**	**98**	**98**	**98**	**98**
	Pos. pred. Value	*84*	76	*83*	69	*81*	NA
	Neg. pred. Value	27	69	59	78	72	**93**
	LR_+_	2	6	6	7	9	NA
Respiratory rate > 55 breaths/min	Diagn. sensitivity	2	10	8	7	16	NA
	Diagn. specificity	**99**	**99**	**99**	**99**	**99**	**99**
	Pos. pred. Value	**92**	**90**	**92**	*80*	**93**	NA
	Neg. pred. Value	27	68	58	76	72	**93**
	LR_+_	4	***17***	***15***	***12***	***30***	NA
Abnormal demeanour	Diagn. sensitivity	*87*	**94**	**97**	**95**	NA	**93**
	Diagn. specificity	**99**	**99**	**99**	**99**	**99**	**99**
	Pos. pred. Value	**100**	**98**	**99**	**97**	**98**	*87*
	Neg. pred. Value	73	**97**	**98**	**98**	**100**	**99**
	LR_+_	***79***	***85***	***88***	***86***	***91***	***85***
Colic	Diagn. sensitivity	3	21	10	2	10	14
	Diagn. specificity	**100**	**100**	**100**	**100**	**100**	**100**
	Pos. pred. Value	**100**	**100**	**100**	**100**	**100**	**100**
	Neg. pred. Value	27	71	58	76	70	**94**
	LR_+_	3^1^	***21***^1^	***10***^1^	2^1^	***10***^1^	***14***^1^
Arched back	Diagn. sensitivity	18	12	3	13	28	36
	Diagn. specificity	**100**	**100**	**100**	**100**	**100**	**100**
	Pos. pred. Value	**100**	**100**	**100**	**100**	**100**	**100**
	Neg. pred. Value	31	69	57	78	74	**95**
	LR_+_	***18***^1^	***12***^1^	3	***13***^1^	***28***^1^	***36***^1^
Abdominal guarding	Diagn. sensitivity	54	59	43	61	*81*	**100**
	Diagn. specificity	*80*	*80*	*80*	*80*	*80*	*80*
	Pos. pred. Value	*88*	60	63	50	65	28
	Neg. pred. Value	39	*80*	64	*86*	**90**	**100**
	LR_+_	3	3	2	3	4	5
Bruxism	Diagn. sensitivity	20	15	12	18	25	21
	Diagn. specificity	**100**	**100**	**100**	**100**	**100**	**100**
	Pos. pred. Value	**100**	**100**	**100**	**100**	**100**	**100**
	Neg. pred. Value	31	69	59	79	74	**94**
	LR_+_	***20***^1^	***15***^1^	***12***^1^	***18***^1^	***25***^1^	***21***^1^
Congested scleral vessels	Diagn. sensitivity	79	*89*	35	73	77	79
	Diagn. specificity	71	71	71	71	71	71
	Pos. pred. Value	**90**	66	55	51	61	21
	Neg. pred. Value	49	**91**	53	*87*	*84*	**97**
	LR_+_	3	3	1	3	3	3
Pale mucous membranes	Diagn. sensitivity	7	10	69	3	27	14
	Diagn. specificity	**97**	**97**	**97**	**97**	**97**	**97**
	Pos. pred. Value	*88*	64	**95**	29	*82*	29
	Neg. pred. Value	28	68	*80*	75	74	**94**
	LR_+_	3	4	***25***	1	***10***	5
Rumen motility reduced or rumen atony	Diagn. sensitivity	73	**90**	**92**	**93**	**99**	**93**
	Diagn. specificity	*87*	*87*	*87*	*87*	*87*	*87*
	Pos. pred. Value	**94**	69	*85*	69	78	35
	Neg. pred. Value	54	**96**	**93**	**98**	**99**	**99**
	LR_+_	6	7	7	7	8	7
Rumen atony	Diagn. sensitivity	6	56	45	49	27	36
	Diagn. specificity	**100**	**100**	**100**	**100**	**100**	**100**
	Pos. pred. Value	**100**	**100**	**100**	**100**	**100**	**100**
	Neg. pred. Value	28	*82*	70	*86*	74	**95**
	LR_+_	6	***56***^1^	***45***^1^	***49***^1^	***27***^1^	***36***^1^
At least one foreign body test positive	Diagn. sensitivity	60	21	28	45	58	38
	Diagn. specificity	55	55	55	55	55	55
	Pos. pred. Value	79	19	31	24	35	6
	Neg. pred. Value	34	62	53	76	76	**93**
	LR_+_	1	1	1	1	1	1
PSA and/or BSA on the right side positive	Diagn. sensitivity	17	53	33	47	51	64
	Diagn. specificity	**90**	**90**	**90**	**90**	**90**	**90**
	Pos. pred. Value	*82*	72	71	60	70	32
	Neg. pred. Value	28	79	63	*84*	79	**97**
	LR_+_	2	5	3	5	5	6
Faeces dark or black	Diagn. sensitivity	NA	18	*80*	11	16	21
	Diagn. specificity	**100**	**100**	**100**	**100**	**100**	**100**
	Pos. pred. Value	NA	**100**	**100**	**100**	**100**	**100**
	Neg. pred. Value	27	74	*87*	79	74	**94**
	LR_+_	NA	***18***^1^	***80***^1^	***11***^1^	***16***^1^	***32***^1^

^1^ The calculation of the LR_+_ required the reduction of the diagnostic specificity from 100 to 99%

*PSA* Percussion and simultaneous auscultation, *BSA* Ballottement and simultaneous auscultation, *NA* not applicable

Values between 80 and 89% are in italic

Values between 90 and 100% are in bold

Values of LR_+_ ≥ 10 are in bold-italic

### Heart rate

**Fig. 2:**
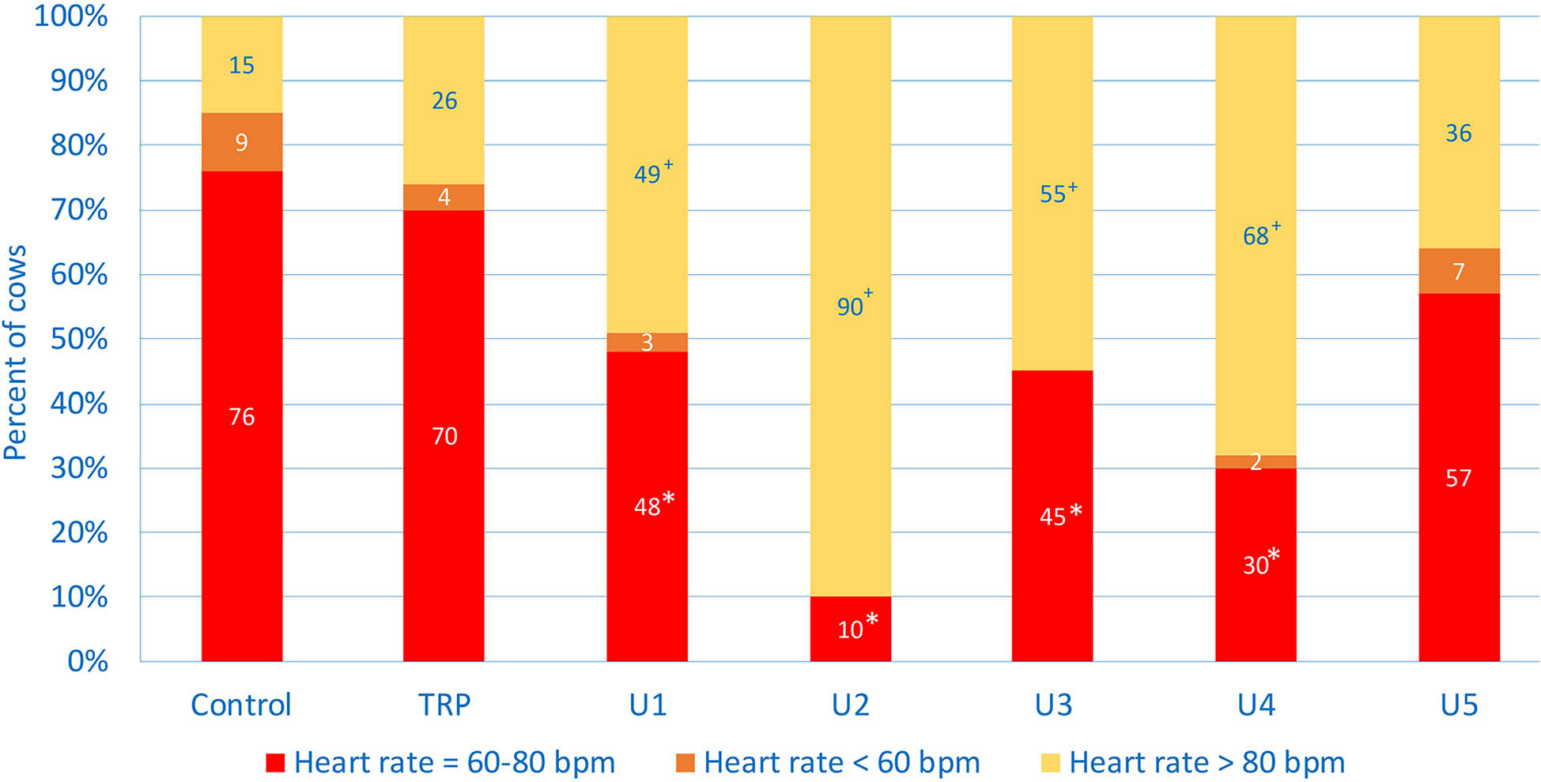
Heart rate in healthy control cows and cows with traumatic reticuloperitonitis (TRP) and type 1 (U1), type 2 (U2), type 3 (U3), type 4 (U4) and type 5 (U5) abomasal ulcer. Frequency distribution of cows with a heart rate within, below or above the reference interval. * Different from percentage of controls in the reference interval, *P* < 0.05, ^+^ Different from percentage of controls above the reference interval, *P* < 0.05

Cows with TRP, U1, U2, U3 and U4 had median heart rates of 76 to 108 beats per min (bpm), which was significantly higher than in controls (72 bpm, Table 1). Seventy-six percent of controls had a heart rate in the reference interval from 60 to 80 bpm (Table 2 and Fig. 2) compared with 48, 10, 45 and 30% in cows with U1, U2, U3 and U4, respectively, which was significantly lower compared with controls. The percentage of control cows with heart rates > 80, > 100 and >  120 bpm were 15, 1 and 1%. Heart rates > 80 bpm were significantly more common in cows with U1 (49%), U2 (90%), U3 (55%) and U4 (68%) than in controls; the difference between cows with U2 and those with other illnesses was significant. The corresponding differences for heart rates of > 100 and >  120 bpm were similar. The LRs_+_ for a heart rate >  100 bmp in cows with abomasal ulcers ranged from 15 to 78 (Table 3), and the positive predictive values for a heart rate >  100 bpm in ill cows ranged from 83 to 98%.

### Respiratory rate

**Fig. 3:**
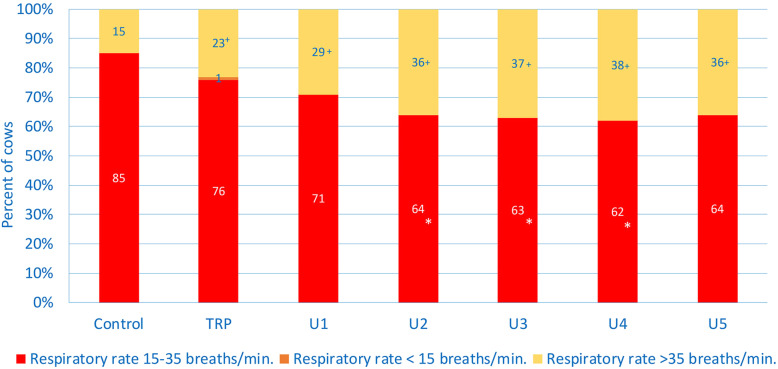
Respiratory rate in healthy control cows and cows with traumatic reticuloperitonitis (TRP) and type 1 (U1), type 2 (U2), type 3 (U3), type 4 (U4) and type 5 (U5) abomasal ulcer. Frequency distribution of cows with a respiratory rate within, below or above the reference interval. * Different from percentage of controls in the reference interval, *P* < 0.05, ^+^ different from percentage of controls above the reference interval, *P* < 0.05

The median respiratory rates of cows with U1, U2 and U4 were significantly higher than those of the controls (Table 1). Eighty-five percent of controls had a respiratory rate in the reference interval from 15 to 35 breaths/min (Table 2 and Fig. 3) compared with 64, 63 and 62% in cows with U2, U3 and U4, which was significantly lower. The percentages of control cows with respiratory rates > 35, > 45 and >  55 breaths/min were 15, 2 and 1%, respectively. Respiratory rates > 35 breaths/min occurred significantly more often in cows with TRP (23%), U1 (29%), U2 (36%), U3 (37%), U4 (38%) and U5 (36%) than in controls. The LRs_+_ for a respiratory rate >  55 breaths/min in cows with U1, U2, U3 and U4 ranged from 12 to 30, and the positive predictive value for all ill cows ranged from 80 to 92% (Table 3).

### Demeanour

The demeanour was abnormal in 1% of the controls (Table 2) compared with 87 to 100% in the ill cows, which was significant. The LR_+_ for abnormal demeanour in ill cows ranged from 79 to 91, and the positive predictive value from 87 to 100% (Table 3).

### Colic

Signs of colic such as restlessness, shifting weight, kicking and a sunken back did not occur in control cows (Table 2) but were seen in cows with U1 (21%), U2 (10%), U4 (10%) and U5 (14%). The LR_+_ for signs of colic in cows with U1, U2, U4 and U5 were 21, 10, 10 and 14, respectively, and the positive predictive value was 100% in all ill cows (Table 3).

### Arched back

An arched back was seen in 0.5% of the control cows (Table 2) and with the exception of cows with U2, was significantly more frequent in the ill cows (TRP 18%, U1 12%, U3 13%, U4 28%, U5 36%). The frequency of arched back was significantly lower in cows with TRP than in cows with U4 and U5. The LR_+_ for arched back exceeded 10 in cows with TRP (18), U1 (12), U3 (13), U4 (28) and U5 (36), and the positive predictive value was 100% in all ill cows (Table 3).

### Abdominal guarding

Abdominal guarding occurred in 20% of controls (Table 2) and was significantly more frequent in ill cows (TRP 54%, U1 59%, U2 43%, U3 61%, U4 81%, U5 100%). The LR_+_ was small with values between 2 and 5 (Table 3).

### Bruxism

Bruxism occurred in 0.5% of controls (Table 2) and was significantly more frequent in ill cows (TRP 21%, U1 15%, U2 12%, U3 18%, U4 25%, U5 21%). The LR_+_ exceeded 10 in all ill cows (TRP 20, U1 15, U2 12, U3 18, U4 25, U5 21) and the positive predictive value was 100% (Table 3).

### Scleral congestion

Scleral congestion occurred in 29% of the controls (Table 2) and significantly more frequently in 73 to 89% of the ill cows with the exception of the cows with U2. The positive predictive value for TRP was 90% (Table 3).

### Mucous membrane colour

Pale mucous membranes occurred in 3% of control cows (Table 2) and significantly more frequently in cows with U2 (69%) and U4 (27%). The LR_+_ for pale mucous membranes was 25 for cows with U2 and 10 for cows with U4, and the predictive value was 95% in cows with U2 (Table 3).

### Rumen motility

**Fig. 4:**
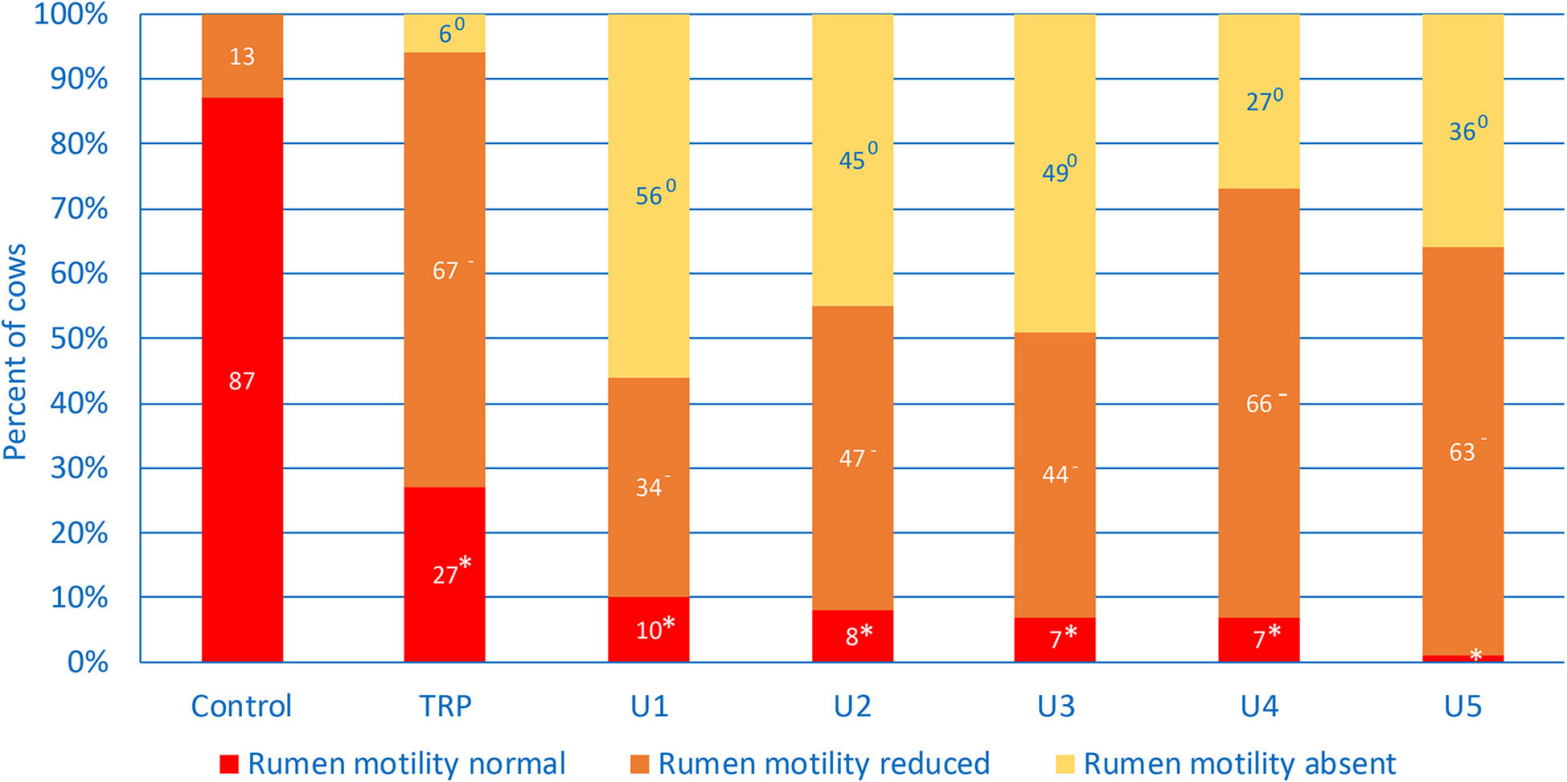
Rumen motility in healthy control cows and cows with traumatic reticuloperitonitis (TRP), type 1 (U1), type 2 (U2), type 3 (U3), type 4 (U4) and type 5 (U5) abomasal ulcer. Frequency distribution of cows with normal, reduced and absent rumen motility. * Different from percentage of controls with normal rumen motility, *P* < 0.05, − different from percentage of controls with reduced rumen motility

Reduced rumen motility occurred in 13% of controls (Table 2 and Fig. 4) and significantly more often in all groups of ill cows. Rumen atony did not occur in controls, and it was significantly less frequent in cows with TRP (6%) than in the other ill cows (U1 56%, U2 45%, U3 49%, U4 27%, U5 36%). The LR_+_ for rumen atony ranged from 27 to 56 for cows with abomasal ulcer and was 6 for cows with TRP (Table 3). The positive predictive value was 100% in ill cows.

### Foreign body tests

At least one foreign body test was positive in 44% of controls (Table 2). This percentage was significantly higher in cows with TRP (60%) and significantly lower in cows with U1 (24%), whereas the other groups did not differ from the controls (U2 28%, U3 45%, U4 58%, U5 39%).

### Percussion and auscultation and ballottement and auscultation on the right side

This procedure produced a positive result in 10% of the controls and significantly more frequently in cows with abomasal ulcer (U1 53%, U2 33%, U3 47%, U4 51%, U5 64%; Table 2). The LR_+_ was lower than 10 in all disease groups (Table 3).

### Faecal colour

Dark to black faeces were not seen in controls (Table 2) and in cows with TRP but occurred in cows with abomasal ulcers; it was significantly more frequent in cows with U2 (80%) than in cows with other ulcer types (11 to 21%). The LR_+_ for dark to black faeces was greater than 10 in cows with abomasal ulcer (U1 18, U2 80, U3 11, U4 16, U5 32), and the positive predictive value was 100% (Table 3).

## Discussion

The reliability indices shown in Table 3 are typically used as measures of laboratory test performance and only rarely for the characterisation of clinical diagnostic procedures. There is only one study that reported the predictive values for abdominal pain in 26 cows with abomasal ulcer [19]. In that study, the diagnostic sensitivity of abdominal pain was 54% and the positive predictive value was 48%. When abdominal pain was combined with the variables haematocrit < 24% and occurrence of occult blood in the faeces, the positive predictive value increased to 100%; however, this was accompanied by a low diagnostic sensitivity of 15%. The present study compared clinical findings of healthy cows and cows with TRP, U1, U2, U3, U4 and U5. To our surprise, even healthy cows had numerous clinical variables with values outside of the reference intervals, but there were also variables that were always or almost always (99.0–99.5%) within the reference interval and thus well suited for distinguishing healthy cows from ill cows. The variables colic, rumen atony and black faeces provided the best selectivity because these clinical signs never occurred in healthy cows; thus cows with these clinical signs are always ill. Other findings that rarely occurred in healthy cows and therefore indicated *illness* were arched back and bruxism (each 0.5%, 1 of 182 cows) and abnormal demeanour, rectal temperature ≤ 38.0 or >  40.0 °C, heart rate >  100 bmp and respiratory rate >  55 breaths per minute (each 1%, 2 of 182 cows). Only mildly abnormal vital signs such as mild hypothermia (38.1–38.5 °C) or hyperthermia (39.1–39.5 °C), slightly increased heart (81–100 bpm) and respiratory rates (36–45 breaths per min) were not suitable as diagnostic criteria because they also occurred in healthy cows. It was surprising that some control cows had abnormal findings. One particular cow had several abnormal signs consecutively but never at the same time. All controls were deemed healthy based on the results as a whole. The fact that some control cows had one abnormal finding emphasizes that differentiation of health and illness cannot usually be based on individual clinical findings but should rather take into account the history and results of all tests. There are numerous physiological causes for these mild deviations including excitement, stress, activity, pregnancy, increased ambient temperature and others [20–22]. Auscultation of reduced rumen motility can be attributable to decreased ingestion of fibre, which is needed to generate sound when it rubs against the rumen mucosa [23]; less fibre leads to diminished sound. Other clinical variables seen in control cows and therefore considered to have poor selectivity included positive percussion and/or ballottement and auscultation (10%), reduced rumen motility (13%), rectal temperature between 39.0 and 39.5 °C (25%), congested scleral vessels (29%) and at least one positive foreign body test (44%). Thus, these findings were not reliable for the differentiation of healthy and ill cows because they commonly occurred in healthy cows. Surprisingly, 44% of the control cows had at least one positive foreign body test even though TRP and other disorders of the cranial abdomen were not present. This calls into question the diagnostic usefulness of foreign body tests.

The demeanour was well suited for the differentiation of healthy and ill cows, and this was supported by LRs_+_ between 79 and 91 in all groups of ill cows. A LR_+_ > 10 is considered high and indicates that the test can be used to rule in the condition in question [18]. The LR_+_ of a test describes how much more likely it is that the test result (or the clinical finding) is from an ill patient than from a healthy patient [18]. Even though demeanour was good for differentiation of healthy and ill cows, it was not useful for differentiation of the various disease conditions because of the high sensitivity at 87 to 97%, which means that a randomly selected ill cow is highly likely to have an abnormal demeanour.

A rectal temperature in the reference interval had little selectivity, whereas a temperature ≤ 38.0 °C was significantly more common in cows with U2 and U4 than in cows with TRP. The low rectal temperature was thought to be associated with shock in cows with U2 and U4. A rectal temperature >  39.5 °C was significantly more common in cows with TRP, U3 and U5 than in cows with U2, but this finding had a relatively low diagnostic sensitivity in all disease groups and was also uncommon in cows with TRP, U3 and U5.

A heart rate > 100 bpm was reliable for differentiation of healthy and ill cows but was only partly useful for differentiation of various disease groups. The diagnostic sensitivity in cows with TRP was only 4% and therefore these cows were unlikely to have a heart rate exceeding 100 bpm. A heart rate > 100 bpm was significantly more frequent in cows with ulcers than in cows with TRP. A heart rate > 100 bpm had a relatively high diagnostic sensitivity only in cows with U2 (62%) and U5 (86%).

Signs of colic were well suited for the differentiation of healthy and ill cows because they did not occur in the former and each had a diagnostic specificity and a positive predictive value of 100%. However, the diagnostic sensitivity of signs of colic in the groups of ill cows was low and ranged from 2 to 21%. This was similar for the variables arched back and bruxism. All these findings (with the exception of arched back in cows with U2) had a high LR_+_ and a low diagnostic sensitivity.

The diagnostic sensitivity of abdominal guarding in 14 cows with U5 was 100% and thus, based on this study, U5 can be ruled out in the absence of a tense abdominal wall. Otherwise, this clinical sign was unsuitable for the differentiation of healthy and ill cows (LR_+_ 2 bis 5) and cows of the different disease groups.

Congestion of scleral vessels had a high diagnostic sensitivity of 73 to 89% for all ill cows except those with U2, but the LR_+_ was low at 1 to 3. Therefore, this clinical sign is not suitable for differentiation of healthy and ill cows or for distinguishing cows with TRP and abomasal ulcer even though at 35% it was significantly less frequent in cows with U2 than in cows of the other disease groups.

Pale mucous membranes had a low diagnostic sensitivity except for cows with U2 (69%) and can aid in differentiating these cows from cows with TRP and other types of abomasal ulcer.

Rumen atony is critical for the differentiation of healthy and ill cows because it did not occur in any of the control cows. Surprisingly, it was significantly less frequent in cows with TRP (6%) than in cows with abomasal ulcer. This was likely because severe changes in demeanour were rarely seen in cows with TRP as opposed to cows with U2 or U4. Marked changes in demeanour significantly affect the gastric centre of the medulla oblongata causing vagal-related problems with rumen motility. This means that cows with complete rumen atony are highly unlikely to have TRP, perhaps because only severe illnesses with inhibitory inputs, such as fever or severe pain acting on the gastric centre in the medulla oblongata, result in complete cessation of rumen motility [24].

Positive foreign body tests were unsuitable for differentiation of healthy and ill cows as well as of cows of the different disease groups even though positive tests were significantly less frequent in cows with U1 and U2 than in cows with TRP. Of note, only 60% of the cows with TRP had at least one positive foreign body test. The most likely reason for this is that many cows referred with TRP have been chronically ill and the clinical signs are less distinct than those seen in acute cases [1, 2]. Of interest, the rate of positive foreign body tests in cows with U4 (58%) and TRP was almost the same and therefore abomasal ulcer must be considered in the differential diagnosis in cows with positive foreign body tests.

Positive percussion and/or ballottement and auscultation on the right side were significantly more frequent in cows with abomasal ulcer than in control cows and cows with TRP, and in cows with U1 than in cows with U2. Overall, a positive test occurred in over 50% of cows with U1, U4 and U5 but was considerably less frequent in control cows and cows with TRP and U2.

Black faeces are a crucial variable for distinguishing cows with U2 from healthy cows or from cows with other types of abomasal ulcer, and in cows with U2 had a high diagnostic sensitivity of 80%. Determination of the haematocrit and testing for occult blood are indicated to assess the severity of anaemia and melena in cows passing black faeces.

## Conclusions

The results of our study showed that multiple clinical findings, such as abnormal demeanour, heart rate > 100 bpm, colic, rumen atony, black faeces, arched back and bruxism, can be used to differentiate healthy cows and those with TRP or abomasal ulcer. Other clinical signs including mild increase in rectal temperature, scleral congestion and a positive reticular foreign body test cannot be used to differentiate healthy cows and those with TRP or abomasal ulcer because they occur in all groups. A detailed history, thorough clinical examination and comprehensive assessment of all available information are needed for a clinical diagnosis.

## Data Availability

The datasets used and analysed for this study are available from the corresponding author on reasonable request.

## Abbreviations

TRP: Traumatic reticuloperitonitis
U1: Abomasal ulcer type-1
U2: Abomasal ulcer type-2
U3: Abomasal ulcer type-3
U4: Abomasal ulcer type-4
U5: Abomasal ulcer type-5
Bpm: Beats per minute
Min: Minute
LR_+_: Positive likelihood ratio
Fig: Figure
